# An epistasis between dopaminergic and oxytocinergic systems confers risk of post-traumatic stress disorder in a traumatized Chinese cohort

**DOI:** 10.1038/s41598-019-55936-8

**Published:** 2019-12-17

**Authors:** Kunlin Zhang, Gen Li, Li Wang, Chengqi Cao, Ruojiao Fang, Shu Luo, Ping Liu, Xiang yang Zhang

**Affiliations:** 1 Laboratory for Traumatic Stress studies and Center for Genetics and BioMedical Informatics Research, CAS Key Laboratory of Mental Health, Institute of Psychology, Chinese Academy of Sciences, Beijing, 100101 China; 2 Department of Psychology, University of Chinese Academy of Sciences, Beijing, 100049 China; 3 Shenzhen Key Laboratory of Affective and Social Cognitive Science, Shenzhen University, Shenzhen, 518060 China; 4 People’s Hospital of Deyang City, Deyang, Sichuan 618000 China

**Keywords:** Genetic interaction, Post-traumatic stress disorder

## Abstract

Post-traumatic stress disorder (PTSD) is a psychiatric syndrome that occurs after trauma exposure. Neurotransmitters such as dopamine and oxytocin have been reported to be involved in neuropathology of PTSD. Previous studies indicated that the dopamine–oxytocin interaction may contribute to behavioral disorders. Thus, exploring the epistasis (gene–gene interaction) between oxytocinergic and dopaminergic systems might be useful to reveal the genetic basis of PTSD. In this study, we analyzed two functional single nucleotide polymorphisms (SNPs), rs2268498 for oxytocinergic gene *OXTR* and rs1801028 for dopaminergic gene *DRD2* based on putative oxytocin receptor–dopamine receptor D2 (OTR–DR2) heterocomplex in a Chinese cohort exposed to the 2008 Wenchuan earthquake (156 PTSD cases and 978 controls). Statistical analyses did not find any single variant or gene–environment interaction (SNP × earthquake-related trauma exposure) associated with provisional PTSD diagnosis or symptoms. An *OXTR*–*DRD2* interaction (rs2268498 × rs1801028) was identified to confer risk of provisional PTSD diagnosis (OR = 9.18, 95% CI = 3.07–27.46 and *P* = 7.37e-05) and further subset analysis indicated that rs2268498 genotypes controlled the association directions of rs1801028 and rs1801028 genotypes also controlled the association directions of rs2268498. Rs2268498 × rs1801028 is also associated with PTSD symptoms (*P* = 0.043). Our study uncovered a genetic and putative function-based contribution of dopaminergic–oxytocinergic system interaction to PTSD.

## Introduction

Post-traumatic stress disorder (PTSD) is a psychiatric disorder that can occur after trauma exposure. Diagnosis of PTSD requires at least six symptoms distributed across four symptom clusters and persisting for at least one month after trauma exposure^1^. With the lifetime prevalence of 5.6% among trauma-exposed population^2^, the development of PTSD is affected by both genetic factors and environment triggers^3–5^. Recent studies also indicated that inherited and acquired risk of genetic and epigenetic have significant contributions to response to trauma exposure^6^. To explore genetic mechanisms of PTSD, many genetic studies have been performed^7^, including the most recent genome-wide association study (GWAS) of Psychiatric Genomics Consortium (PGC)^8^. Most of the PTSD genetic studies have utilized single nucleotide polymorphisms (SNPs) as markers, and focused on main effects or gene–environment interaction effects of single gene variant; however, gene–gene interaction (epistasis) has seldom been explored. Here gene–gene interaction is defined as that the effect of one locus genotype on a phenotype depends on the genotype at other loci^9^. Since interactions between loci contribute to the biological and biochemical pathways that underpin disease, detecting their interactions will be useful to elucidate the genetic mechanisms underlying the complex diseases such as PTSD^9–11^. Testing genetic variants of correlated genes that are involved in the same pathway or interact with each other is a feasible strategy for gene–gene interaction analysis^9^, with reduced searching space and increased statistical power.

The neurotransmitters, such as dopamine (DA), oxytocin (OXT) and serotonin, are endogenous chemicals that transmit signal across a synapse between two nerve cells^12^. DA plays important roles in neuropsychological processes including motivation, executive functions, reward and stress^13–15^. Abnormal dopaminergic signaling have been revealed to be associated with several mental disorders, including attention deficit hyperactivity disorder (ADHD), drug and alcohol dependence, schizophrenia and PTSD^16–19^. OXT is needed for childbirth and is involved in regulations of both social behaviors (such as social bonding, sexual activity and motivation) and nonsocial behaviors (such as brain development, feeding and memory)^20^. OXT has been reported to be associated with schizophrenia, autism spectrum disorders and PTSD^21–23^. Particularly, the oxytocin receptor gene (*OXTR*) polymorphism may predict PTSD through interaction with attachment style^24^. It has been reported that OXT–DA interactions affect social behavior like motivation^25^. Furthermore, previous studies indicated that abnormal OXT–DA interactions may contribute to behavioral disorders such as autism, sexual dysfunction, addiction and depression^26^. The mechanism of their interactions has been investigated for several years and the results indicated that dopamine receptor D2 (DR2) encoded by dopaminergic gene *DRD2* and oxytocin receptor (OTR) encoded by oxytocinergic gene *OXTR* may directly work together, by forming OTR–DR2 heteromer^27,28^.

There have been some candidate gene association studies on PTSD with *DRD2* and *OXTR* respectively; the results of *DRD2* were not consistent (only 11 out of total 20 studies showed significant results)^7^ and the results of *OXTR* were quite a few^24,29^. Furthermore, *DRD2* and *OXTR* are co-expressed in multiple tissues of normal human brain according to the results of gene co-expression search engine SEEK^30^. Thus, we hypothesized that the gene–gene interaction between the two genes at genetic variant level may be involved in the PTSD development. There are two SNPs, rs2268498 in *OXTR* and rs1801028 (Ser311Cys) in *DRD2*, are quite meaningful and important. Rs2268498 (risk allele C) is localized in the promoter flanking region of the *OXTR* gene, which was reported to correlated to negative emotionality^31^ that is one of the factors of PTSD^1,32,33^; this SNP also affects the gene expression of *OXTR*^34^. Rs1801028 (risk allele C) is a non-synonymous SNP of *DRD2*, which has been frequently studied and known to be associated with schizophrenia^35,36^ which shares genetic risk with PTSD^8^. Rs1801028 has been investigated in our study before while no significant result was obtained^37^; rs2268498 has not been investigated in PTSD yet. In this study, to investigate our hypothesis, we analyzed the *OXTR* SNP rs2268498 and the *DRD2* SNP rs1801028 in a traumatized Chinese cohort and examined the association between the gene–gene interaction and provisional PTSD diagnosis.

## Results

### Association between *OXTR*–*DRD2* and provisional PTSD diagnosis

The demography of all subjects is present in Supplementary Table 1. The genotyping call rate for both SNPs is 100%. The minor allele frequencies (MAFs) of rs2268498 are 0.2724 in cases and 0.316 in controls respectively; the MAFs of rs1801028 are 0.03846 in cases and 0.03681 in controls respectively (Supplementary Table 2). The Hardy-Weinberg equilibrium (HWE) test results are also listed in Supplementary Table 2. For provisional PTSD diagnosis inferred from PCL-5, there were no significant main effects for single gene-based analysis (*P* > 0.05), either without or with gene–environment interaction (SNP × earthquake-related trauma exposure) included in the logistic regression model (Supplementary Table 2). Gene–gene interaction analysis showed that rs2268498 × rs1801028 interaction was associated with provisional PTSD diagnosis (odds ratio (OR) = 9.18, 95% confidence interval (CI) = 5.25–16.05, *P* = 7.37e-05. Table 1 shows more detailed information). The association signals came from both females and males (*P* = 0.000494 and 0.0200 respectively); the effect size (OR) of the interaction was much larger in males than that in females (OR = 62.51 and 7.60 respectively) (See Supplementary Tables 3 and 4 for more details).

**Table 1:** Summary of logistic regression model of rs2268498 × rs1801028 for PTSD diagnosis.

Variable	OR (95% CI)	beta	Std. Error	*t* value	*P* value	*P*_perm_
rs2268498 × rs1801028	9.18 (3.07, 27.46)	2.21660	0.55917	3.964	7.37e-05	1e-06
rs2268498	0.72 (0.53, 0.98)	−0.32803	0.15877	−2.066	0.0388	0.03824
rs1801028	0.17 (0.04, 0.66)	−1.80092	0.70395	−2.558	0.0105	0.008239
Gender	1.45 (0.94, 2.22)	0.37017	0.21795	1.698	0.0894	—
Age	1.08 (1.05, 1.10)	0.07557	0.01174	6.438	1.21e-10	—
Trauma exposure	1.15 (1.04, 1.28)	0.14235	0.05401	2.636	0.0084	—
Depression symptoms	1.12 (1.10, 1.15)	0.11397	0.01146	9.947	2.599e-23	—

***P***_perm_, permutation *P* value of SNP-related variable.

### Rs2268498 and rs1801028 controlled association for each other

**Figure 1:**
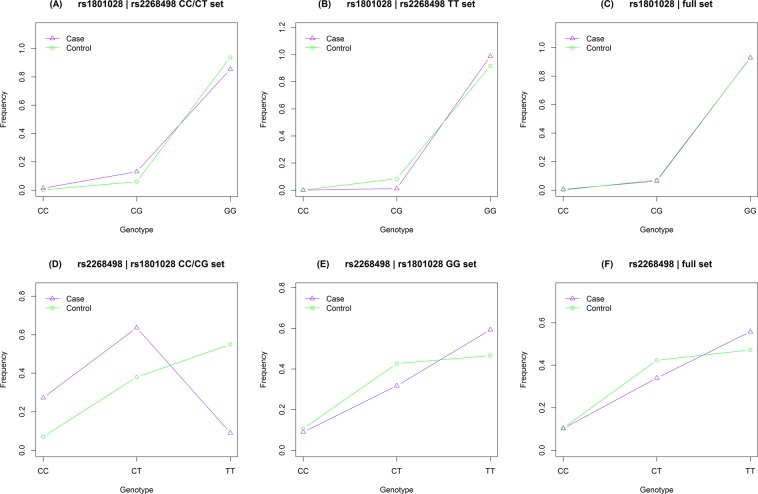
Genotype frequency variability between case and control. (**A–C**) For rs1801028 in the rs2268498 CC/CT set, the rs2268498 TT set and the full set, respectively. (**D–F**) For rs2268498 in the rs1801028 CC/CG set, the rs1801028 GG set and the full set, respectively.

Figure 1 shows genotype frequency variability between case and control subjects, respectively for the subsets obtained based on genotype and the full set. In Fig. 1(A–C) are for rs1801028 and (D–F) are for rs2268498. When we divided samples by rs2268498 genotypes CC/CT and TT, rs1801028 minor allele C was associated with increased risk of provisional PTSD diagnosis (OR = 3.02, 95% CI = 1.32–6.94, *P* = 0.009163) in rs2268498 CC/CT set and with decreased risk of provisional PTSD diagnosis (OR = 0.10, 95% CI = 0.01–0.78, *P* = 0.02863) in rs2268498 TT set (Table 2). Rs2268498 was associated with decreased risk of provisional PTSD diagnosis (OR = 0.71, 95% CI = 0.52–0.97, *P* = 0.03287) in the rs1801028 GG set (Table 3). The sample size of genotype subsets in females or in males was too small to present, although the association trends were consistent with those of overall samples (details not shown). All results of subset analysis were confirmed by permutation analysis (Tables 2 and 3). Thus, rs2268498 and rs1801028 controlled association with provisional PTSD diagnosis for each other.

**Table 2:** Logistic regression analysis of rs1801028 (with minor allele C) in the rs2268498 CC/CT set, the rs2268498 TT set and the full set, respectively.

rs2268498 genotype	Sample size (case/control)	MAF (case/control)	OR (95% CI)	*P* value	*P*_perm_
CC/CT	585(69/516)	0.07971/0.03198	3.02 (1.32, 6.94)	0.009163	0.006785
TT	549(87/462)	0.005747/0.04221	0.10 (0.01, 0.78)	0.02863	0.01621
ALL	1134(156/978)	0.03846/0.03681	1.03 (0.53, 2.04)	0.922	0.9754

***P***_perm_, permutation *P* value of SNP-related variable.

**Table 3:** Logistic regression analysis of rs2268498 (with minor allele C) in the rs1801028 CC/CG set, the rs1801028 GG set and the full set, respectively.

rs1801028 genotype	Sample size (case/control)	MAF (case/control)	OR (95% CI)	*P* value	*P*_perm_
CC/CG^a^	82(11/71)	0.5909/0.2606	13.06 (2.52, 67.73)	0.002215	0.0003993
GG	1052(145/907)	0.2483/0.3203	0.71 (0.52, 0.97)	0.03287	0.03264
ALL	1134(156/978)	0.2724/0.316	0.85 (0.64, 1.14)	0.2825	0.2831

***P***_perm_, permutation *P* value of SNP-related variable.

^a^The sample size is too small, thus the results are with low confidence.

### Association between *OXTR*–*DRD2* and PTSD symptoms

For PTSD symptoms, no significant main effects or G × E effects was found (Supplementary Table 5). The G × G *OXTR–DRD2* is associated with PTSD severity (rs2268498 × rs1801028: beta = 3.43, 95% CI = 0.11–6.75, *P* = 0.043). See Table 4 for detailed information. The association signal is not strong and both females and males contribute to the association signal (See Supplementary Tables 6 and 7 for more details). Subset analysis did not obtain any significant result (details are not shown).

**Table 4:** Summary of linear regression model of rs2268498 × rs1801028 for PTSD symptoms.

Variable	Beta (95% CI)	Std. Error	*t* value	*P* value	*P*_perm_
rs2268498 × rs1801028	3.43 (0.11, 6.75)	1.6931	2.0255	0.0430482	0.043476
rs2268498	−0.26 (−1.21, 0.69)	0.4865	−0.5353	0.592562	0.591971
rs1801028	−2.16 (−5.35, 1.03)	1.6291	−1.3251	0.1854098	0.185062
Gender	2.76 (1.43, 4.10)	0.6826	4.0477	5.53e-05	—
Age	0.33 (0.27, 0.39)	0.0321	10.2257	1.57e-23	—
Trauma exposure	1.41 (1.07, 1.76)	0.1770	7.9908	3.30e-15	—
Depression symptoms	0.79 (0.72, 0.86)	0.0372	21.2928	8.20e-85	—

***P***_perm_, permutation *P* value of SNP-related variable.

### *OXTR*–*DRD2* in different age groups

Our results showed that for provisional PTSD diagnosis, rs2268498 × rs1801028 is more significant in young adults defined as subjects with age <50 years old (OR = 9.24 and *P* = 0.00470) than in old adults defined as subjects with age > = 50 years old (OR = 6.76 and *P* = 0.0226); for PTSD symptoms, rs2268498 × rs1801028 is significant in young adults (beta = 5.11 and *P* = 0.0129) but not in old adults (beta = −1.08 and *P* = 0.7183). Supplementary Table 8 showed the details.

## Discussion

Through epistasis (gene–gene interaction) analysis, our study identified the *OXTR*–*DRD2* (rs2268498 × rs1801028) interaction to be associated with provisional PTSD diagnosis and PTSD symptoms. Interestingly, rs2268498 and rs1801028 controlled each other’s association directions for provisional PTSD diagnosis. The interaction signals came from both females and males. The two SNPs have been investigated in PGC-PTSD GWAS^8^ and we listed the results in Supplementary Table 9. All the association test *P* values (with different gender and ethnic group combinations) in PGC-PTSD GWAS are far from genome-wide significance (5e-8) or suggestive significance (e-5), indicating that the two SNPs are not associated with PTSD directly and our G × G analysis provides feasibly new information. It needs to mention that there is about six times as many controls as cases in our study (978:156), which just reflects the provisional PTSD prevalence in our epidemiologic samples and is feasible.

Our results indicated that the G × G (rs2268498 × rs1801028) effects are different between young adults (age < 50 years old) and old adults (age > = 50 years old) (Supplementary Table 8). In our data, age is significantly correlated to both provisional PTSD diagnosis (OR = 1.07 and *P* = 4.72e-10, by logistic regression analysis) and PTSD symptoms (beta = 0.32 and *P* = 3.24e-23, by linear regression analysis). Furthermore, a two-sided fisher’s exact test showed a distribution difference of provisional PTSD diagnosis cases between two age groups (*P* = 3.24e-8) and a two-sided *t*-test showed a distribution difference of PTSD symptoms between the two age groups (t = −5.46 and *P* = 5.97e-8). Thus, the effect of age on PTSD may contribute to the differences of G × G effects between different age groups.

The OXT–DR2 interaction was supported by evidence from animal experiments of male rats^28^. Our study showed that the statistical interaction exists in both women and men. In details, the association of females is more significant than that of males, which may due to the larger samples size of females than males; the interaction effect size of males is much larger than that of females, showing that males may be more sensitive to the interaction. In summary, the interaction looks like generally applies for both females and males. *OXTR* and *DRD2* have been separately reported to be correlated with stress response through gene expression or DNA methylation which may regulate gene expression. For *OXTR*, it was reported that *OXTR* expression significantly increased in the amygdala in response to chronic social instability stress for adult female rats^38^; DNA methylation of *OXTR* in human blood is with dynamic changes after acute psychosocial stress^39^. For *DRD2*, down-regulated *DRD2* gene expression in striatum is correlated to stress-induced depression-like behaviors in rats^40^; alterations of human dopaminergic genes including *DRD2* are associated with low resilience to stress^41^. Our statistical genetics finding further indicated that there is a gene-gene interaction between the two genes in the context of stress response.

Rs1801028 (G > C) is corresponding to the amino acid residue polymorphism S311C of human DRD2 protein and both SIFT^42^ and PolyPhen-2^43^ predicted that S311C may be with deleterious impact to DR2 (Supplementary Table 10). Together with the function of the *OXTR* SNP rs2268498 (influencing *OXTR* expression)^34^, it is possible to infer the SNP–SNP interaction to impact biological function level. It has been found that at protein level, DR2 and OTR interacts with each other by forming heterocomplex. Our study found that a functional genetic variant interaction of rs2268498 × rs1801028 might confer risk of provisional PTSD diagnosis. The further subset analysis showed that when rs2268498 C allele corresponding to high *OXTR* gene expression^34^ presented, rs1801028 C allele (unstable DRD2 protein) increased PTSD risk. When rs1801028 GG genotype (stable DRD2 protein) presented, rs2268498 C allele (high *OXTR* gene expression) decreased PTSD risk. This may be explained that high level OTR and unstable DRD2 are difficult to form feasible heterocomplex, which may lead to dynamic disequilibrium of OTR–DRD2 heteromer, influencing PTSD development. This provides evidence that dopaminergic–oxytocinergic interaction may be involved in behavior/mental disorders^26^, which deserves further investigation in other mental disorders. Furthermore, besides OTR–DRD2 heteromer, there are many G protein-coupled receptor (GPCR) heteroreceptor complexes involved in brain network^44^. Our results suggest a possibility that these heteroreceptor complexes may be replicated in PTSD, as well as in other mental disorders. Still, since genetic control of gene expression can differ between blood and brain^45^, and our results were based on blood samples, all these inferences need to be carefully considered.

There are several limitations in our study. First, PTSD diagnosis was based on PCL-5, a self-report measurement, other than clinician-administered instruments, which will need to be used in future studies. Second, only one trauma type- exposure to the 2008 earthquake was examined. Our results would need to be confirmed in other trauma-related PTSD cohorts. Third, the sample size of our study is modest in comparison to other genetic studies. Moreover, the male sample size is relatively smaller than that of the female’s (due to the female-male gender ratio of our epidemiologic samples). Hopefully, a much larger sample with balanced sexes could be used to replicate our findings in future. Also, there is no information about pre-earthquake trauma exposure or some conditions to which PTSD symptoms may be attributed (such as substance use, bereavement, etc.) and PTSD symptoms which may preclude the conclusion that PTSD is developed after the earthquake; still, causality can be inferred given that genes inherently serve as the first temporal variable. In future study, we will take the information into account.

Our study firstly identified the *OXTR*–*DRD2* interaction at genetic variant level to be significantly associated with a higher PTSD risk based on provisional PTSD diagnosis. Since the genetic variants correspond to polymorphisms in mRNA or protein level, the genetic interaction provides a possibility of existence of functional interaction, suggesting that abnormal interaction of dopaminergic and oxytocinergic systems may be a vulnerable factor for PTSD development. Of course, we should note these interpretations were based on provisional PTSD diagnosis. In summary, our gene–gene interaction results may provide new viewpoints in terms of the biological mechanisms and genetic architectures of PTSD, which will be helpful for early diagnosis and prevention.

## Materials and Methods

### Study sample

The study samples we used are epidemiologic samples which are feasible for generalization^46^. In detail, all the subjects were recruited from Hanwang Town, Mianzhu City, China. In the 2008 Wenchuan earthquake, the town was almost destroyed. Five and a half years after the earthquake, we conducted the survey in a large rebuilt community of the town. Subjects were predominantly Chinese adults (more than sixteen years old) who survived the earthquake. All of them were without a major psychiatry history or mental retardation such as schizophrenia and organic mental disorders^47,48^. We assessed PTSD symptoms by the PTSD Checklist for DSM-5 (PCL-5)^49,50^, a reliable and repeatable 20-item self-reported scale to capture the PTSD symptoms of DSM-5. The items of PCL-5 particularly focus on the earthquake-related trauma and the Chinese version of PCL-5 has been used for earthquake-exposure Chinese before^32^. We instructed participants to complete the PCL-5 referring to the “Wenchuan Earthquake”. The likely DSM-5 PTSD diagnosis was then inferred based on PCL-5, including at least one intrusion symptom, one avoidance symptom, two negative alterations in cognitions and mood symptoms, and two arousal symptoms endorsed as 2 or greater. Earthquake-related trauma exposure was considered as environment factor and was measured by a ten-question scale. The sum score was calculated by adding 10 items (0: not experienced or 1: experienced for each item) as the level of trauma exposure. The details of the ten questions have been described before^51^. Also, we measured depression symptoms with the Center for Epidemiological Studies-Depression (CES-D) Scale^52^. We then collected peripheral blood samples and extracted DNA for genotyping by using a standard phenol-chloroform protocol^53^. For each sample, at least 120 ng DNA was used for genotyping experiment. A total of 1134 subjects (156 PTSD cases and 978 controls) with complete clinical information, and genotyping data were included in our study.

### SNP genotyping and quality control

We investigated rs2268498 in oxytocinergic gene *OXTR* and rs1801028 (Ser311Cys) in dopaminergic gene *DRD2*. We conducted genotyping by using a custom-by-design 2 × 48-Plex SNPscan^TM^ Kit (Genesky Biotechnologies Inc., Shanghai, China), which is based on a multiplex fluorescence PCR and a double ligation. The PCR primers were showed in Supplementary Table 11. To assess genotyping accuracy, we randomly chose 10% samples for replication by the same platform. The concordance was 100%, showing that the genotyping is accurate. A Hardy-Weinberg equilibrium (HWE) test was performed by exact tests with efficient computational methods^54,55^. For quality control (QC), we excluded subjects with missing call rate > 0.2, and removed SNPs with call rate < 0.95 or HWE test *P* < 0.05 for controls or MAF (minor allele frequency) < 0.01 in controls. No sample or SNP was removed for the quality control.

### Statistical analyses

All of our statistical analyses were performed by using PLINK v1.09^55^ and R3.4.4 (https://cran.r-project.org/). We first analyzed the association for provisional PTSD diagnosis. For a single variant-based analysis, we employed an additive model of SNP minor allele count per subject in a logistic regression with gender, age, depression symptoms (CES-D scale score, the possible confounding factor) and trauma exposure as covariates. The gene–environment interaction (SNP × trauma exposure) was further included. The results indicated that the gene–environment interaction was not significant, thus it was not included in following analyses. For gene–gene interaction analysis, we utilized an additive model of minor allele count per subject in a logistic regression with predictors SNP1, SNP2, SNP1 × SNP2, gender, age, depression symptoms and trauma exposure. To explore the possible different association properties between females and males, we also carried on the gene–gene interaction analysis for females and males, respectively. We then investigated the association for PTSD symptoms (PCL-5 total score which is with rang 0–80 and treated as a continuous variable) by using the similar methods as above, except that we employed linear regression instead of logistic regression.

To explore the detailed gene–gene interaction property, we employed subset analysis. We divided all samples into two subsets according to rs2268498 genotype: the CC/CT set (samples with C alleles) and the TT set (samples with genotype TT). Then we performed regressions (with gender, age, depression symptoms and trauma exposure as covariates) for rs1801028 respectively in the two subsets. Correspondingly, we stratified all samples by rs1801028 genotype (CC/CG and GG), examining the association for rs2268498 respectively.

In our analysis, age is significant factor affecting provisional PTSD diagnosis. The age of our study subjects is in a wide range (16–73) while the median of age is 48 and more than 50% of the subjects are with ages in the much narrower range (42, 56) (Supplementary Table 1 and Supplementary Fig. 1). To explore possible different association property between diverse age groups, we divided out samples into young adults (age < 50 years old) and old adults (age > = 50 years old). We then performed association test to detecting G × G (rs2268498 × rs1801028) effect for the two groups respectively.

All the *P* values of the regressions in our analyses are two-sided. Threshold of *P* value is considered as 0.05. We employed a permutation test with 1,000,000 cycles for SNP-related variables to adjust for possible bias due to the small sample size.

### Statements of ethical approval, accordance and informed consent

The Institutional Review Board of Institute of Psychology, Chinese Academy of Sciences approved this study. All study procedures involving human participants were in accordance with the national and institutional research committee’s ethical standards. All participants signed informed consents. Among them, two were under the age of 18 years and signed informed consents with permission of their legal guardians who also provided written informed consents.

## Supplementary information

Supplementary materials

## Data Availability

Supplementary materials are available online.
