# Supplementary material for: An epistasis between dopaminergic and oxytocinergic systems confers risk of post-traumatic stress disorder in a traumatized Chinese cohort
Source: Sci Rep. 2019 Dec 17;9:19252. doi: 10.1038/s41598-019-55936-8 (PMC6917732; doi:10.1038/s41598-019-55936-8)
Supplement: Supplementary file 1 — Supplementary materials [file 41598_2019_55936_MOESM1_ESM.pdf]

Supplementary materials

An epistasis between dopaminergic and oxytocinergic systems confers risk of post-traumatic stress disorder in a traumatized Chinese cohort

Kunlin Zhang [1,2], Gen Li [1,2], Li Wang [1,2,*], Chengqi Cao [1,3], Ruojiao Fang [1,2], Shu Luo [4], Ping Liu [4], Xiangyang Zhang [1,2]

[1] Laboratory for Traumatic Stress studies and Center for Genetics and BioMedical Informatics Research, CAS Key Laboratory of Mental Health, Institute of Psychology, Chinese Academy of Sciences, Beijing 100101, China

[2] Department of Psychology, University of Chinese Academy of Sciences, Beijing 100049, China

[3] Shenzhen Key Laboratory of Affective and Social Cognitive Science, Shenzhen University, Shenzhen, China

[4] People's Hospital of Deyang City, Deyang, Sichuan, China

* **Corresponding author:** Institute of Psychology, Chinese Academy of Sciences, 16 Lincui Road, Beijing 100101, China. Telephone: +86-10-6483-6985. Fax: +86-10-6483-6985. E-mail address: wangli1@psych.ac.cn (L. Wang).

Running Head: Dopaminergic–oxytocinergic interaction in PTSD

Supplementary Table 1 Demography of samples in our study.

Data set	No. of samples (female/male)	Range of age	1st Quartile of age	Median of age	3rd Quartile of age	Age of samples (mean±sd)
Case	156 (115/41)	21-66	46	53	60	52.53±8.59
Control	978 (658/320)	16-73	41	47	55	47.37±9.98
Total	1134 (773/361)	16-73	42	48	56	48.08±9.96

Supplementary Table 2 Results of single SNP-based analysis for PTSD diagnosis.

SNP	Allele[a]	MAF (case/control/all)	HWE test *P* (case/control/all)	Effect[b]	OR (95% CI)	*P* value	P_{perm}
rs2268498	C/T	0.2724/0.316/0.31	0.07233/0.5064/0.2117	M	0.85 (0.64, 1.14)	0.2825	0.2831
				MG	0.94 (0.50, 1.75)	0.8364	0.8371
				GE	0.98 (0.84, 1.13)	0.74	0.74
rs1801028	C/G	0.03846/0.03681/0.03704	0.1972/1/0.6639	M	1.03 (0.53, 2.04)	0.922	0.9754
				MG	1.13 (0.27, 4.69)	0.8679	0.8697
				GE	0.98 (0.68, 1.40)	0.8923	0.894

MAF, minor allele frequency. G×E: gene–environment interaction (SNP×trauma exposure).

P_{perm}, permutation *P* value of SNP-related variable.

[a] minor/major.

[b] M: main effect of SNP based on logistic regression without G×E; MG: main effect of SNP based on logistic regression with G×E; GE: results for G×E based on logistic regression with G×E.

Supplementary Table 3 Summary of the logistic regression model of rs2268498×rs1801028 in females for PTSD diagnosis.

Variable	OR (95% CI)	beta	Std. Error	*t* value	*P* value	P_{perm}
rs2268498×rs1801028	7.60 (2.43, 23.78)	2.02787	0.58203	3.484	**0.000494**	**2e-06**
rs2268498	0.71 (0.48, 1.03)	-0.34639	0.19276	-1.797	0.072338	0.07171
rs1801028	0.21 (0.05, 0.86)	-1.56460	0.72227	-2.166	0.030293	0.02185
Age	1.09 (1.06, 1.12)	0.08476	0.01358	6.241	4.36e-10	-
Trauma exposure	1.13 (0.99, 1.27)	0.11839	0.06342	1.867	0.061931	-
Depression symptoms	1.12 (1.09, 1.15)	0.11163	0.01402	7.962	1.70e-15	-

P_{perm}, permutation *P* value of SNP-related variable.

Supplementary Table 4 Summary of the logistic regression model of rs2268498×rs1801028 in males for PTSD diagnosis.

Variable	OR (95% CI)	beta	Std. Error	*t* value	*P* value	P_{perm}
rs2268498×rs1801028	62.51 (1.92, 2038.29)	4.13532	1.77783	2.326	**0.0200**	**0.0005401**
rs2268498	0.73 (0.41, 1.27)	-0.31974	0.28563	-1.119	0.2630	0.2658
rs1801028	0.01 (0.00, 1.80)	-4.35195	2.52112	-1.726	0.0843	0.0553
Age	1.05 (1.00, 1.10)	0.04783	0.02424	1.973	0.0485	-
Trauma exposure	1.21 (0.98, 1.49)	0.18885	0.10629	1.777	0.0756	-
Depression symptoms	1.12 (1.08, 1.17)	0.11702	0.02013	5.814	6.11e-09	-

P_{perm}, permutation *P* value of SNP-related variable.

Supplementary Table 5 Results of single SNP-based analysis for PTSD symptoms.

SNP	Allele[a]	MAF	Effect[b]	Beta (95% CI)	Std. Error	*t* value	*P* value	P_{perm}
rs2268498	C/T	0.3100	M	-0.0043 (-0.93,0.92)	0.4703	-0.0092	0.9926	0.992524
			MG	-0.27 (-2.14,1.60)	0.9539	-0.2807	0.779	0.780912
			GE	0.078 (-0.40,0.56)	0.2452	0.3175	0.7509	0.767817
rs1801028	C/G	0.0370	M	0.15 (-2.13,2.43)	1.1645	0.1298	0.8968	0.896739
			MG	0.41 (-4.07,4.89)	2.2853	0.1793	0.8577	0.858359
			GE	-0.081 (-1.28,1.12)	0.6129	-0.1316	0.8954	0.901327

MAF, minor allele frequency. G×E: gene–environment interaction (SNP×trauma exposure).

P_{perm}, permutation *P* value of SNP-related variable.

[a] minor/major.

[b] M: main effect of SNP based on logistic regression without G×E; MG: main effect of SNP based on linear regression with G×E; GE: results for G×E based on linear regression with G×E.

Supplementary Table 6 Summary of the linear regression model of rs2268498×rs1801028 in females for PTSD symptoms.

Variable	Beta (95% CI)	Std. Error	*t* value	*P* value	P_{perm}
rs2268498×rs1801028	3.33 (-0.41, 7.08)	1.9124	1.7436	0.081633	0.081401
rs2268498	-0.41 (-1.57, 0.74)	0.5895	-0.6973	0.48583	0.485978
rs1801028	-1.37 (-5.01, 2.27)	1.8576	-0.7383	0.46058	0.460625
Age	0.37 (0.29, 0.44)	0.0376	9.7905	2.09E-21	-
Trauma exposure	1.61 (1.19, 2.03)	0.214	7.5252	1.48E-13	-
Depression symptoms	0.73 (0.64, 0.82)	0.0455	15.9529	1.11E-49	-

P_{perm}, permutation *P* value of SNP-related variable.

Supplementary Table 7 Summary of the linear regression model of rs2268498×rs1801028 in males for PTSD symptoms.

**Variable**	**Beta (95% CI)**	**Std. Error**	***t* value**	***P* value**	**P_{perm}**
rs2268498×rs1801028	4.45 (-2.65, 11.55)	3.6216	1.2288	0.219965	0.214072
rs2268498	-0.16 (-1.85, 1.52)	0.8603	-0.1892	0.850063	0.849918
rs1801028	-4.34 (-11.00, 2.33)	3.4006	-1.2762	0.202728	0.198018
Age	0.23 (0.10, 0.35)	0.0618	3.6483	0.000304	-
Trauma exposure	0.95 (0.33, 1.58)	0.3179	2.9959	0.002929	-
Depression symptoms	0.91 (0.78, 1.03)	0.0647	14.0479	6.16E-36	-

P_{perm}, permutation *P* value of SNP-related variable.

Supplementary Table 8 Summary of association test for G×G (rs2268498×rs1801028) effect in different age groups.

**Age group** [a]	**Phenotype**	**Sample size** [b]	**OR/Beta (95% CI)** [b]	***P* value**	**P_{perm}**
Young adults	Provisional PTSD diagnosis	55/578	9.24 (1.98, 43.19)	0.00470	0.000194
Old adults	Provisional PTSD diagnosis	101/400	6.76 (1.31, 34.95)	0.0226	0.011844
Young adults	PTSD symptoms	633	5.11 (1.09, 9.12)	0.0129	0.015097
Old adults	PTSD symptoms	501	-1.08 (-6.95, 4.79)	0.7183	0.71795

[a] Young adults: age < 50 years old. Old adults: age >=50 years old.

[b] case/control for phenotype provisional PTSD diagnosis and single number for phenotype PTSD symptoms.

[c] OR is for phenotype Provisional PTSD diagnosis and Beta is for phenotype PTSD symptoms.

P_{perm}, permutation *P* value of SNP-related variable.

Supplementary Table 9 Summary of association test results (with different gender and ethnic group combinations) in PGC-PTSD GWAS for rs2268498 and rs1801028.

SNP	Allele 1	Allele 2	Cohort	OR/Beta [a]	SE	*P*	Direction
rs2268498	T	C	All	0.0087	0.0302	0.7731	--+-+-+?+--+-++?+--
			EA	-0.0077	0.0432	0.8594	--+-+-+?+
			AA	0.0349	0.0447	0.435	--+-++?
			F	0.96831	0.0412	0.4348	Null
			M	1.04477	0.0498	0.3798	Null
			FEA	0.91119	0.0632	0.1408	Null
			MEA	1.05961	0.071	0.4148	Null
			FAA	1.04071	0.0565	0.4801	Null
			MAA	1.02912	0.076	0.7059	Null
rs1801028	C	G	All	-0.0471	0.1045	0.652	+--++-+-+--+-+-?+?-
			EA	-0.0895	0.1327	0.5003	+--++-+-+
			AA	0.0043	0.1844	0.9813	--+-+-?
			F	0.8101	0.1554	0.1755	Null
			M	1.30109	0.1692	0.1198	Null
			FEA	0.60908	0.2184	0.02319	Null
			MEA	1.51831	0.2114	0.04827	Null
			FAA	1.09341	0.2288	0.6963	Null
			MAA	0.89521	0.3438	0.7474	Null

All, all samples; EA, European American; AA African American; F, females; M, males; FEA, female European American; MEA, male European American; FAA, female African American; MAA, male African American. Null, not applicable. [a] OR is for F, M, FEA, MEA, FAA and MAA; Beta is for All, EA and AA.

Supplementary Table 10 Predictions of rs1801028 impact to protein by comparative genomics analysis (SIFT and PolyPhen-2).

**SNP**	**Amino acid change**	**SIFT prediction**	**SIFT score**	**PolyPhen-2 prediction**	**PolyPhen-2 score**
rs1801028 (G>C)	S311C	Damaging	0.01 (for 311C)	Probably damaging	0.992

SIFT dbSNP rsIDs (based on NCBI's dbSNP (build 132) database). PolyPhen-2 v2.2.2r398.

Supplementary Table 11 PCR primers for genotyping by using SNPscan™ Kit.

**SNP**	**P1**	**Seq 1**	**P2**	**Seq 2**	**P3**	**Seq 3**
rs2268498	rs2268498_CR	TGCCTTCATCCAGCCGTAGG	rs2268498_TR	TGCCTTCATCCAGCCGTCGA	rs2268498_3R	GTGAGGCAGGGGTGTTTTACCT
rs1801028	rs1801028_GR	CTGACTCTCCCCGACCCATC	rs1801028_CR	CTGACTCTCCCCGACCCATG	rs1801028_3R	CCACCAYGGTCTCCACAGCA

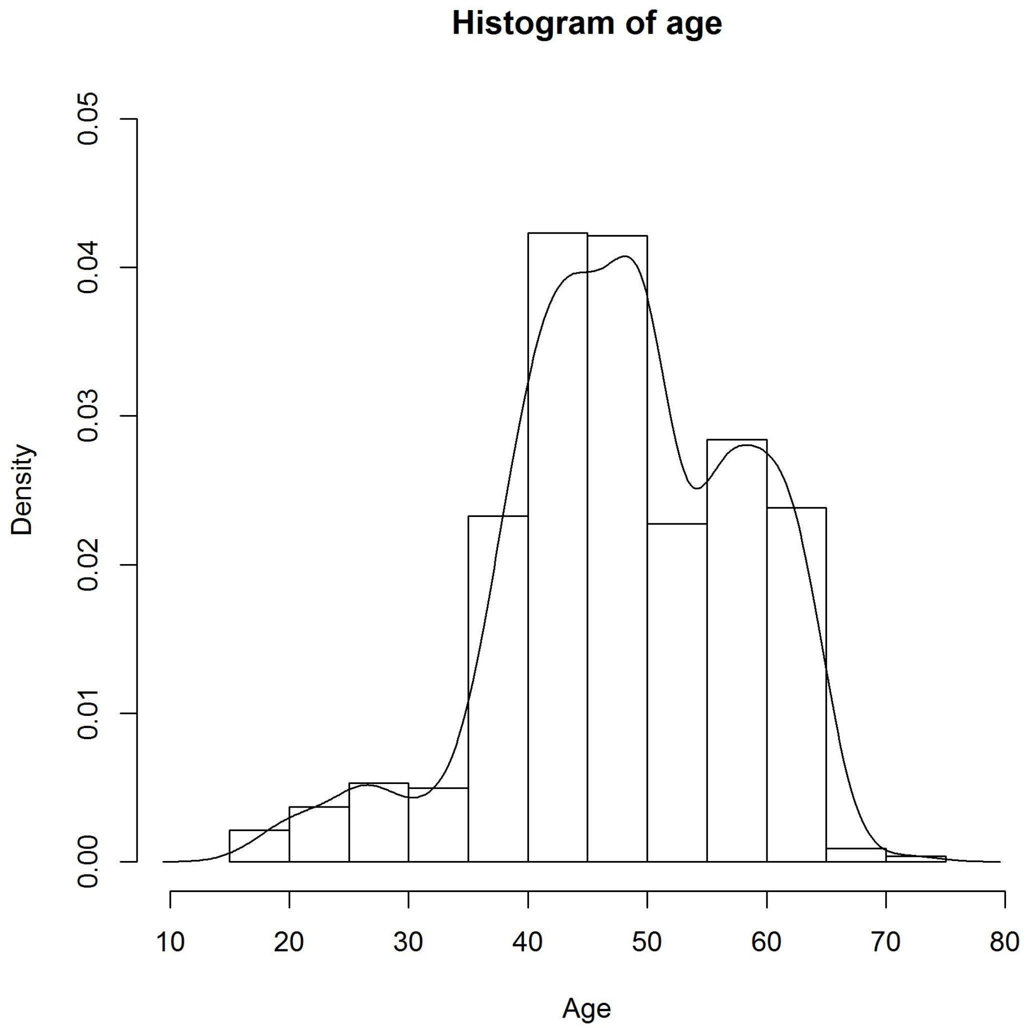

Supplementary Figure 1 Histogram and density plot of age.
